# Sjogren Syndrome-Associated Autonomic Neuropathy

**DOI:** 10.7759/cureus.25563

**Published:** 2022-06-01

**Authors:** Nourhan Chaaban, Timothy Shaver, Shilpa Kshatriya

**Affiliations:** 1 Internal Medicine, University of Kansas School of Medicine-Wichita, Wichita, USA; 2 Rheumatology, University of Kansas School of Medicine-Wichita, Wichita, USA; 3 Cardiology, Heartland Cardiology, Wichita, USA; 4 Cardiology, University of Kansas School of Medicine-Wichita, Wichita, USA

**Keywords:** xerostomia, xerophthalmia, acute motor and sensory axonal neuropathy (amsan), autonomic neuropathy, anti-ro/ssa, primary sjogren syndrome (pss)

## Abstract

Sjogren syndrome (SS) is one of the common autoimmune diseases characterized by dryness of the eyes and mouth described as “xerophthalmia” and “xerostomia,” respectively. Affected patients may also experience complex neurological symptoms characterized by extremity paresthesia and pain as well as dizziness and presyncope. In fact, patients may present initially with pure neurological symptoms. Thus, the diagnosis of SS remains a clinical challenge. We report a case here of a patient with primary Sjogren syndrome (pSS) experiencing severe autonomic neuropathy symptoms.

A 53-year-old female patient diagnosed with pSS at the age of 30 years presented with a four-month history of palpitations and dizziness. A tilt table test showed positive findings with significant symptomatic orthostatic hypotension. The patient was started on midodrine therapy followed by a beta-blocker with improvement in her symptoms. There was also augmentation of her SS disease management by introducing IV IgG therapy.

The diagnosis of neuropathy in SS is complex and requires a combination of clinical signs and symptoms. This case report focuses on the neurological manifestations of SS, especially the ones related to autonomic neuropathy. We aim to share awareness of the neurological manifestations of patients with pSS and alert physicians that it could be the initial presentation of this systemic disease.

## Introduction

Sjogren’s syndrome (SS) is a systemic autoimmune disease that mainly affects the exocrine glands and usually presents as persistent dryness of the mouth and eyes due to functional impairment of the salivary and lacrimal glands [1]. It is a complex and heterogeneous disease potentially leading to disability and quality of life impairment [2]. It can be associated with different organ-specific autoimmune diseases, such as thyroiditis, primary biliary cirrhosis, or cholangitis [3]. In the absence of an associated systemic autoimmune disease, patients with this condition are classified as having primary SS [4]. According to the 2016 American College of Rheumatology (ACR)/European League Against Rheumatism (EULAR) classification criteria [5], the diagnosis of pSS requires an individual fulfilling the inclusion criteria with a summed score of greater or equal to 4. The clinical features of SS can be divided into broad categories of exocrine glandular features and extra-glandular features [6]. The following case focuses on the extra-glandular features of primary SS, specifically its neurological manifestations. The diagnosis of SS remains a clinical challenge. In this study, we report a case of a patient with primary Sjogren syndrome (pSS) experiencing severe autonomic neuropathy symptoms characterized by dizziness and palpitations.

## Case presentation

**Figure 1:**
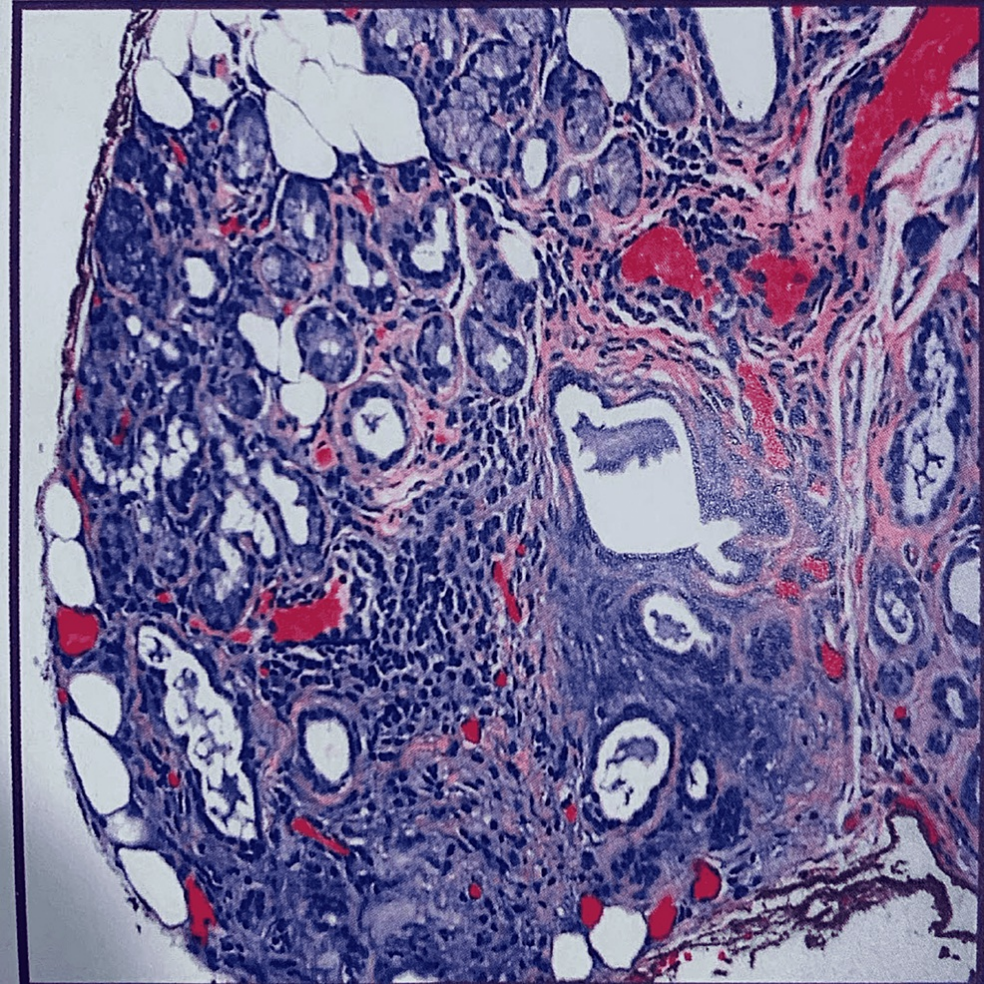
Lip biopsy: minor salivary glands with approximately one focus of lymphoplasmacytic infiltration per 2 sq/mm (one focus score), compatible with Sjogren’s syndrome

A 53-year-old female patient was referred by her primary care physician to our cardiology practice with symptoms of dizziness and palpitations. She has been diagnosed with Grave’s disease and Sjogren syndrome. Regarding her SS, she had negative antibody testing, and it was confirmed on lip biopsy (Figure 1). She has been maintained on hydroxychloroquine therapy and follows regularly with rheumatology.

Her palpitations were worse with exertion and were described as “funny beats.” It happens weekly with associated dyspnea. She reported that two months prior, she had an episode of syncope when she was standing on the porch. She reported prodromal symptoms of dizziness before her syncope episode, which was witnessed by her husband. At that time, she visited the emergency department and had an unremarkable workup. Since that episode, she continued to experience further episodes of dizziness while sitting but mostly while assuming an erect posture. Prior to the last few months, she had never sustained previous symptoms of dizziness or syncope. On review of systems, the patient reports generalized fatigue. She also reported persistent pain described as a stabbing sensation, mostly in the lower extremities and to a lesser extent in the hands with associated paresthesia. No associated fever or chills. The initial electrocardiogram showed a normal sinus rhythm of a heart rate of 60 bpm with non-specific ST and T wave abnormalities (Table 1).

**Table 1:** Lab results with unremarkable findings SSA: Sjogren syndrome-related antigen A; SSB: Sjogren syndrome type B.

Lab Parameter (Units)	Value	Reference Range
Thyroid-stimulating hormone (TSH) (uIU/mL)	3.5	0.45-4.5
Cortisol level AM (mcg/dL)	12.4	6.2-19.4
Erythrocyte sedimentation rate (ESR) (mg/L)	7	0-20
C-reactive protein (CRP) (mg/L)	2.5	0-4.9
Anti-Ro/SSA antibodies (U/mL)	4	<10
Anti-LA/SSB Antibodies (U/mL)	3	<10

**Figure 2:**
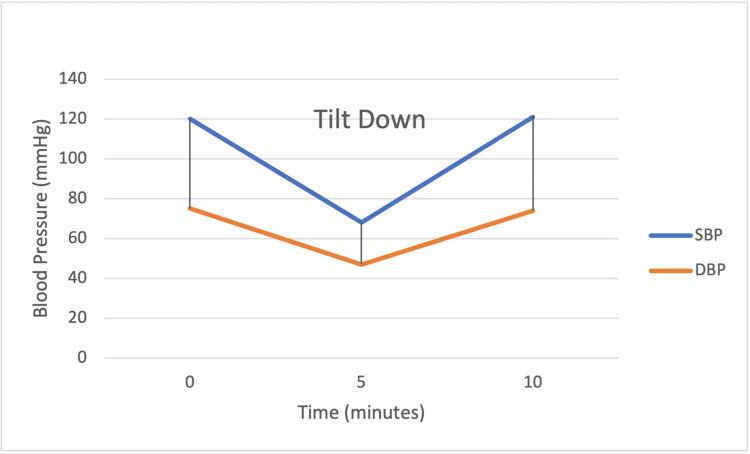
Tilt table test SBP: Systolic blood pressure; DBP: Diastolic blood pressure.

A Holter monitor (Figure 2) was placed for 24 hours. The patient remained in sinus rhythm. There were 234 premature ventricular contractions (PVCs) with one ventricular pair and no ventricular runs. There were 17 premature atrial contractions (PACs) with no atrial pairs and no atrial runs. The minimum heart rate was 62 bpm, and the maximum heart rate was 134 bpm with an average heart rate of 88 bpm. The stress echocardiogram was normal with no evidence of ischemia, average exercise capacity, and normal left ventricular systolic function with an estimated ejection fraction of 60%-65%.

The tilt table test was performed with results that suggested orthostatic hypotension/autonomic dysfunction. Baseline blood pressure prior to tilting was 120/75 mmHg with a heart rate (HR) of 73 bpm. Upon tilting within five minutes, the patient’s blood pressure dropped to 68/47 mmHg with an HR of 64 associated with symptoms of dizziness. There was no frank syncope, but blood pressure promptly improved in the supine position with a discharge BP of 121/74 mmHg and HR of 72 bpm.

We encouraged salt intake and prescribed midodrine 5 mg twice a day, given her continued symptoms and dramatic orthostasis. The patient was also started on digoxin 250 mcg a day for PVCs. Further workup was performed. She had nerve conduction studies that suggest small nerve fiber neuropathy. She then had a skin biopsy by her rheumatologist from the left calf that demonstrated a reduced epidural nerve fiber density of 0.69 units (< 4.3 is considered abnormal). Hence, we intensified the SS treatment, and the patient was started on IV IgG therapy. The patient was seen after two months with improvement to complete resolution of her symptoms.

## Discussion

Neurological symptoms occur in approximately 20% of patients with SS and might be the presenting manifestations of the disease [7]. It is a common characteristic of extra-glandular manifestation of pSS. It is variable and classified into three board categories including the central nervous system, peripheral neuropathies, and autonomic nervous system. [8]. The pathogenesis for the phenotype of each disease varies depending on the type of lesion, and in some instances, it remains unclear.

Peripheral neuropathy is a major neurological manifestation of SS, with an etiology being vasculitis in the peripheral nerves, similar to those seen in other collagen vascular diseases [9]. It is likely the most common neurologic complication and is identified in 2%-25% of primary SS patients [10]. It is a wide spectrum that could be characterized as sensorimotor or painful sensory polyneuropathy, sensory ataxic neuropathy, trigeminal neuropathy, autonomic neuropathy, multiple neuropathies, polyradiculoneuropathy, and multiple cranial neuropathies [11,12,9,13].

One of the most common types of peripheral neuropathies in pSS is axonal sensory and sensorimotor polyneuropathies [10,14,15]. In this type, patients usually report asymmetrical distal extremity paresthesia [14]. Physical examination would reveal decreased sensation to light touch and vibration with mild weakness. One of the distinctive features here is the low complement levels and cryoglobulinemia seen in laboratory testing [14]. On the other hand, small fiber neuropathy is the second most common neurological presentation. Patients present with distal extremity painful sensation described as burning or shock-like paresthesia [16]. Also, a mild subset of patients may report some autonomic symptoms characterized by an unusual change in bowel habits, micturition, and infrequently sweating episodes. Examination shows decreased sensation to pinprick stimuli and temperature with preserved motor strength. There is also a reduction of anti-Ro/SSA antibody levels in this group of patients [17].

Autonomic neuropathy is a subset of peripheral neurological manifestations in patients with pSS. It is one of the forms that can rarely present solely and may occur in association with a sensory ataxic neuropathy [18]. Its pathogenesis is related to autonomic reaction and dysfunction rather than actual destruction and inflammation of the autonomic nerves. In fact, it has been suggested that cholinergic dysfunction is the hallmark of dysautonomia in pSS [19]. It is clinically characterized by alterations in the parasympathetic autonomic system. In one study, patients with primary SS showed signs of both sympathetic and parasympathetic dysfunction, especially those with anti-Ro/SSA and anti-La/SSB antibodies, and had an abnormal blood pressure reaction to tilt compared with controls [19]. Patients report abnormal cardiovascular reflex testing such as orthostatic hypotension, abdominal pain, diarrhea, and abnormalities in micturition in addition to decreased lacrimation and salivation. Mandl et al. described these findings with a reported lower expiration to inspiration ratio and a higher degree of blood pressure drop in response to orthostasis [19].

Patients with symptoms and signs suggesting neuropathy generally should proceed with electromyography (EMG)/nerve conduction studies [20]. The diagnosis of small fiber neuropathy is based on demonstrating decreased density of epidermal nerve fibers in a skin biopsy from the affected region [17]. The management of peripheral neuropathy manifestations is complex with no definitive cure. It is a primarily symptomatic treatment and stabilization. To focus on the autonomic neuropathy subtype, fludrocortisone, midodrine, or beta-blockers might add a benefit to the symptomatic patients. Interestingly, severe forms could be managed with either IVIG [21] or rituximab.

Our patients reported fatigue associated with dizziness. The tilt table test was positive revealing remarkable orthostatic hypotension. Thus, our patient would be classified as a patient experiencing autonomic neuropathy in pSS. She was started on midodrine with an intensification in SS disease management. She showed improvement in her symptoms. She was then maintained on metoprolol 50 mg twice per day for associated tachycardia. On subsequent follow-ups, the patient reported resolving symptoms with improved quality of life and daily functioning.

## Conclusions

Patients diagnosed with pSS can present in outpatient clinical settings with various neurological symptoms including loss of sensation or lower extremity pain or paresthesia as well as dizziness and syncope. Most of these patients are misdiagnosed or misinterpreted. Practitioners should appreciate the possibility of extra-glandular symptoms of SS as this certainly affects management. In this case, our focus was to highlight the peripheral nervous clinical manifestations in patients with pSS. We aimed to share awareness of the presence of neurological manifestations in patients with pSS and alert physicians that this could be the initial presentation of this systemic disease. Thus, research should be conducted to investigate the association of autonomic dysfunction with primary SS further.
